# The promise of incretin-based pharmacotherapies for metabolic dysfunction-associated fatty liver disease

**DOI:** 10.1007/s12072-025-10795-6

**Published:** 2025-03-26

**Authors:** Harendran Elangovan, Jenny Elizabeth Gunton, Ming Hua Zheng, Jian-Gao Fan, George Boon Bee Goh, Henning Gronbaek, Jacob George

**Affiliations:** 1 Storr Liver Centre, Westmead Institute for Medical Research, The University of Sydney, Sydney, NSW Australia; 2 Department of Gastroenterology and Hepatology, Westmead Hospital, Westmead, NSW Australia; 3 Centre for Diabetes, Obesity and Endocrinology (CDOE), Westmead Institute for Medical Research, The University of Sydney, Sydney, NSW Australia; 4 Faculty of Medicine and Health, The University of Sydney, Sydney, NSW Australia; 5 Department of Diabetes and Endocrinology, Westmead Hospital, Westmead, NSW Australia; 6 Department of Hepatology, MAFLD Research Centre, The First Affiliated Hospital of Wenzhou Medical University, Wenzhou, China; 7 Centre for Fatty Liver Disease, Department of Gastroenterology, Xinhua Hospital Affiliated to Shanghai Jiao Tong University School of Medicine, Shanghai, China; 8 Department of Gastroenterology and Hepatology, Singapore General Hospital, Singapore, Singapore; 9 Duke-NUS Medical School, Singapore, Singapore; 10 Department of Hepatology and Gastroenterology, Aarhus University Hospital, Aarhus, Denmark; 11 Department of Clinical Medicine, Aarhus University, Aarhus, Denmark

**Keywords:** MAFLD, MASH, Steatohepatitis, Cardiovascular–kidney–liver–metabolic axis, GLP1, GIP, Glucagon

## Abstract

**Background:**

The presence of excess liver fat secondary to metabolic dysregulation represents the end-organ manifestation of a systemic disease that can progress to steatohepatitis, cirrhosis and its feared complications of clinical decompensation and hepatocellular cancer. Since metabolic dysfunction-associated fatty liver disease (MAFLD) is highly prevalent globally, there is a pressing need to augment lifestyle interventions with pharmacotherapies to ameliorate disease burden and reduce adverse liver-related events.

**Purpose:**

This review summarises current evidence for the utility of incretin mimetics in the MAFLD/MASH arena.

**Methods:**

A literature review that encompassed multiple database searches to inform the evidence base for incretin drugs in MAFLD/MASH.

**Results:**

Incretin mimetics demonstrate multifarious benefits across the metabolic diseases spectrum with mounting evidence for their role in remitting steatohepatitis and liver fibrosis. Weight loss and insulin sensitisation contribute, but additional mechanisms may also be engaged. Gastrointestinal adverse effects are common but for most, can be managed while preserving the hepatic and cardiometabolic benefits.

**Conclusion:**

The literature reveals benefits from incretin-based therapies for MASH, but data on whether they improve long-term hepatic outcomes are awaited to support their future incorporation into routine clinical care.

## MAFLD and MASH: the scale of the problem

Metabolic dysfunction associated fatty liver disease (MAFLD) is the most common liver disease affecting up to a third of the global population [1]. The incidence of MAFLD and its progressive subtype, metabolic dysfunction associated steatohepatitis (MASH) is projected to increase, in line with the symbiotic relationship between liver fat infiltration, metabolic syndrome and insulin resistance, the molecular hallmark of disease pathophysiology. Consistent with this notion, liver disease progression is associated not only with an increased risk of liver-related adverse outcomes from hepatic decompensation and hepatocellular carcinoma (HCC), but also increased all-cause, cardiovascular and extrahepatic cancer-related mortality, which are the leading causes of death in patients with MAFLD/MASH [2]. Indeed, growing recognition of the steatotic liver diseases as a major public health concern and evolving research paradigms have led to updated iterations of both the European and more recently, Chinese guidelines with the expressed goals to positively influence the natural history of disease and prevent negative clinical outcomes [3, 4].

To date, the cornerstone of MAFLD/MASH management has focused on lifestyle interventions that converge on weight loss through net negative energy balance, improved dietary quality and composition, increased physical activity (both aerobic and resistance), and reduced sedentary behavior. MASH resolution and fibrosis regression is directly proportional to the degree of weight loss achieved by patients, with > 10% bodyweight reduction suggested to be effective in achieving significant improvements in liver fat content, inflammatory activity and fibrosis [5]. While refinements in exercise and diet quality improves hepatic fat and their benefits for cardiometabolic health are proven, definitive studies with the accepted gold standard of liver biopsy are lacking. Moreover, lifestyle modification while elegant in concept, is difficult to recapitulate in everyday clinical practice. This stems from the sociocultural, psychologic, and biologic adaptations stemming from longstanding excess adiposity that can make such lifestyle interventions unacceptable and often an unsustainable prospect. This is despite acceptance by the community of the manifest benefits of lifestyle modification to health and wellbeing. Hence, there is a pressing need to develop new therapeutic approaches to complement the role of lifestyle intervention to ameliorate the progression of metabolic diseases in their broadest sense.

Complimenting the long overdue shift in philosophy to conceptualize metabolic dysfunction as a cabal of multisystem comorbidities driven by diet-induced obesity and insulin resistance of which MAFLD is but one manifestation, there has been growing interest in nurturing and/or repurposing therapies that may have multipronged benefits upon the herein-labeled cardiovascular-kidney-liver-metabolic (CKLM) axis. Incretin-based therapies which have generated significant enthusiasm in recent years may fulfill this pressing clinical need and will be the focus of this review.

## Outline of the incretin class of drugs

The incretin effect refers to the potentiation of meal stimulated insulin secretion through the action of gut-derived glucagon-like peptide-1 (GLP1) and glucose-dependent insulinotropic polypeptide (GIP) on pancreatic islet β-cells. These glucoregulatory effects are attenuated in type 2 diabetes mellitus (T2DM) and restoration of this action through the application of exogenous receptor agonism provides effective glycemic control. The incretin analogs have established efficacy in the management of T2DM and are sanctioned as guideline directed medical therapy for this condition.

**Fig. 1:**
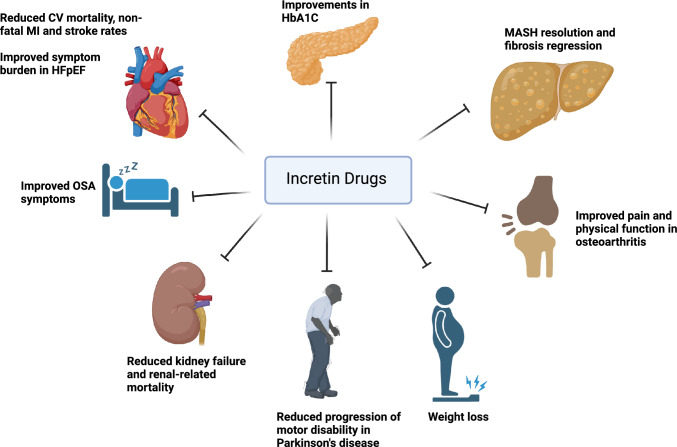
Multiple benefits of using incretin analogs on organ physiology

Similarly, incretin-mimetics also modulate appetite and reward pathways and have demonstrated utility as potent weight loss agents, another major clinical indication for use. Mathematical modeling of weight loss kinetics suggests that incretin drugs attenuate appetite-feedback control mechanisms by up to 70% accounting for their relative longevity compared with dietary modification approaches in facilitating weight loss [6]. Newer agents like retatrutide, a GLP1/GIP/glucagon receptor tri-agonist, have produced weight reductions of up to 24.2% body weight after 48 weeks (the Retatrutide Phase 2 Obesity Trial), approaching equivalence with bariatric surgical procedures [7]. Collectively, these medications provide important adjuncts for cardio-metabolic risk factor control. Later work has pivoted to assessing their impact in attenuating disease expression across the CKLM axis Fig. 1.

**Fig. 2:**
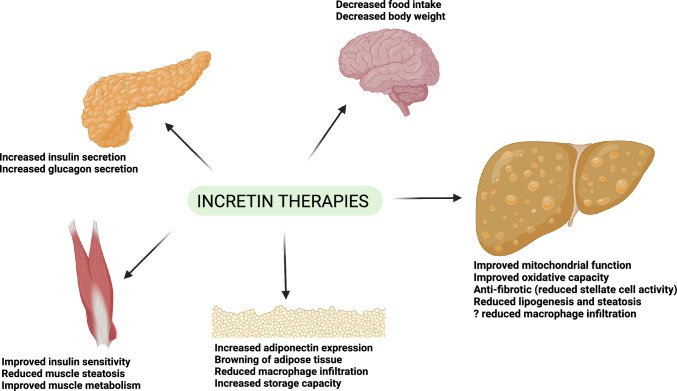
The proposed end organ effects of incretin pharmacology in promoting MASH resolution

Since 2016, there have been several large randomized clinical trials (RCTs) that have shown the benefit of GLP1 receptor agonists in reducing the incidence of death from cardiovascular causes, non-fatal myocardial infarction, and non-fatal stroke [8–10]. Incretin drugs also demonstrated benefit in improving functional limitations and quality of life in patients with heart failure with preserved ejection fraction (HFpEF), an entity for which until recently there has been a shortage of treatment options [11, 12]. Importantly, the benefits for end-organ function appear above and beyond what would be expected [13] from risk factor mitigation (e.g., improvements in HbA1C, blood pressure, serum lipids, and weight etc.) alone. It has been speculated that incretin analogs may engage additional mechanisms (i.e., anti-inflammatory, vasculo-protective etc.) that could account for their observed clinical efficacy. Multiple phase 2 and 3 trials support the use of incretin drugs in chronic kidney disease (CKD) [14], osteoarthritis [15], obstructive sleep apnea (OSA) [16] and even in early Parkinson’s disease [17], demonstrating the pleiotropic benefits of these agents in preventing chronic disease progression (Fig. 2).

A summary of the major clinical studies is highlighted in Table 1. Intriguingly, signals from retrospective data suggest that the risk modifier effect of these agents can be recapitulated in the primary prevention setting as well [18].

**Table 1:** A summary of phase 3 randomized clinical trial data of the incretin drugs across the CKLM disease spectrum

*Type 2 diabetes mellitus (T2DM)*		
SUSTAIN programme [19−20]	Once weekly subcut Semaglutide (0.5 or 1 mg)	Semaglutide at both doses, produced meaningful reductions in Hba1c and weight loss across the T2DM continuum. Comparators were placebo (SUSTAIN 1, 5 and 6), sitagliptin (SUSTAIN 2), exenatide extended release (ER) (SUSTAIN 3), insulin (SUSTAIN 4), and dulaglutide (SUSTAIN 7)
PIONEER programme [25−26]	Semaglutide oral once daily (3, 7 or 14 mg)	Oral Semaglutide provided dose dependent superior HbA1C reductions compared with placebo (PIONEER 1), empagliflozin (PIONEER 2), sitagliptin (PIONEER 3 and 7) and similar HbA1C reductions as liraglutide (PIONEER 4). The drug was safe in moderate renal impairment (PIONEER 5). 7 and 14 mg doses were more effective in inducing weight loss of at least 5% compared with placebo
SURPASS 1 and 2 (2021) [32, 33]	Once weekly subcut Tirzepatide (5, 10 or 15 mg)	Tirzepatide at all doses was superior to placebo and Semaglutide for HbA1c reduction. In SURPASS 2, Tirzepatide was superior to Semaglutide 1 mg for weight loss
*Obesity*		
STEP Programme [34−35]	Once weekly subcut Semaglutide (2.4 mg)	Semaglutide was superior to placebo in reducing weight in overweight/obese patients (STEP 1), with T2DM (STEP 2), as an adjunct to intensive behavior therapy (STEP 3), producing sustainable weight loss effects with continued use (STEP 4 and 5), demonstrated effectiveness in east Asian populations (STEP 6 and 7) and teenagers (STEP TEENS), promoting superior weight loss compared with Liraglutide (STEP 8) and in reversing pre-diabetes (STEP 10)
SURMOUNT Programme [7, 44–46]	Once weekly subcut Tirzepatide (10 or 15 mg)	Tirzepatide at all doses provided significant and sustained reductions in bodyweight compared with placebo (SURMOUNT 1) (5/10/15 mg doses), in patients with T2DM (SURMOUNT 2), synergy with lifestyle interventions (SURMOUNT 3) and sustained weight loss with continued use (SURMOUNT 4)
Cardiovascular disease (CVD)		
LEADER (2016) [8]	Once daily subcut Liraglutide (dose ≥ 1.8 mg) vs placebo	Liraglutide reduced the rates of death from cardiovascular causes, non-fatal myocardial infarction, or non-fatal stroke among patients with type 2 diabetes mellitus
REWIND (2019) [9]	Once weekly subcut Dulaglutide (1.5 mg) vs placebo	Dulaglutide reduced the rates of death from cardiovascular causes, non-fatal myocardial infarction, or non-fatal stroke among patients with type 2 diabetes mellitus
SELECT (2023) [10]	Once weekly subcut Semaglutide (2.4 mg) vs placebo	Semaglutide reduced the rate of death from cardiovascular causes, non-fatal myocardial infarction, or non-fatal stroke among overweight/obese patients without T2DM
STEP-HFpEF (2023) [11] and STEP-HFpEF DM (2024) [12]	Once weekly subcut Semaglutide (2.4 mg) vs placebo	Semaglutide improved symptom burden and physical function in obese patients with and without T2DM with HFpEF
SUMMIT (2024) [47]	Once weekly subcut Tirzepatide (15 mg) vs placebo	Tirzepatide led to lower rates of the composite outcome of death from cardiovascular events and worsening of heart failure events in patients with HFpEF and obesity
*Chronic kidney disease (CKD)*		
FLOW (2024) [14]	Once weekly subcut Semaglutide (1 mg) vs placebo	Semaglutide reduced the risks of major kidney events and death from cardiovascular causes in patient with T2DM and CKD
*MASH*		
ESSENCE (2024) [48]	Once weekly subcut Semaglutide (2.4 mg) vs placebo	Semaglutide was superior to placebo in remitting steatohepatitis and promoting fibrosis regression
*Osteoarthritis*		
STEP-9 (2024) [15]	Once weekly subcut Semaglutide (2.4 mg) vs placebo	Semaglutide reduced body weight and pain related to knee osteoarthritis and improved physical function
*Obstructive sleep apnea (OSA)*		
SURMOUNT-OSA (2024) [16]	Once weekly subcut Tirzepatide (10 or 15 mg) vs placebo	Tirzepatide reduced the apnea–hypopnea index, body weight, CRP, blood pressure and improved sleep-related patient outcomes

*T2DM* Type 2 diabetes mellitus, *HFpEF* Heart failure with preserved ejection fraction, *CKD* Chronic kidney disease, *OSA* Obstructive sleep apnea, *subcut* subcutaneous injection

## Incretin-based therapies in MAFLD/MASH

Direct action of GLP1 and GIP agonism on liver tissue is controversial [49]. Beyond risk factor mitigation with glycemic control and weight management which is the major mechanism by which these drugs treat MAFLD/MASH, lesser benefits have been speculated to be related to the upregulation of adiponectin, off-target alterations in hepatic fat metabolism and attenuation of inflammation. The application of direct glucagon receptor agonism on the liver may also provide additional benefits. There is a need for further pre-clinical work to identify the full gamut of biologic mechanisms by which incretin pharmacotherapies might alter hepatic pathophysiology. This would have important pharmacokinetic considerations as well as guide the optimal combination of agonism approach to apply in the clinical context. Some of the proposed mechanisms of incretin drugs in modulating hepatic physiology are summarized in Fig. 2.

Liver benefits ascribed to the use of GLP1 agonists were first suggested with reductions in liver fat content, a proxy marker for early MASH resolution [50]. Given the uncertainties around the non-invasive grading of MASH, later trials have emphasized histologic correlation. The LEAN study was the first trial to show superiority of Liraglutide over placebo in resolving biopsy-proven MASH and ameliorating fibrosis progression [51]. Similarly, Newsome et al. compared once daily subcutaneous Semaglutide (0.1 mg, 0.2 mg, and 0.4 mg) to placebo in 320 patients with MASH and stage F1-3 fibrosis. Variable doses of Semaglutide were superior to placebo in remitting MASH but not in improving hepatic fibrosis [52]. More recently, the results of part 1 of the ESSENCE study, a phase 3, 240-week, double-blinded placebo-controlled trial in adults with MASH and stage F2-3 fibrosis who were randomized to receive either once weekly subcutaneous Semaglutide 2.4 mg or placebo has been presented. In this largest trial of GLP1 agonists in MASH to date, efficacy analysis (which included the first 800 patients) demonstrated that once weekly Semaglutide was superior to placebo in reducing steatohepatitis without worsening of fibrosis (62.9% vs 34.1%, estimated difference in responder proportions (EDP) 28.9% [95% CI 21.3; 36.5], p < 0.001) and fibrosis improvement by ≥ 1 stage without worsening of steatohepatitis (37% vs 22.5%, EDP 14.4% [95% CI 7.5; 21.4], p < 0.001) at the 72-week time point. Significantly more patients with MASH and F2/3 fibrosis treated with Semaglutide achieved resolution of steatohepatitis and improvement in fibrosis compared to placebo (32.8% vs 16.2%, EDP 16.6% [95% CI 10.2; 22.9], p < 0.001) [48].

Dual receptor agonism approaches are also showing promise in phase 2 trials of MASH with both Tirzepatide (a dual GLP1/GIP agonist) and Survodutide (a dual GLP1/Glucagon receptor agonist) meeting their primary end-points of remitting steatohepatitis without worsening of fibrosis and improvement in fibrosis stage of ≥ 1 grade without worsening of steatohepatitis when compared with placebo [53, 54]. Table 2 summarizes the key findings.

**Table 2:** Primary outcomes from Tirzepatide (SYNERGY-NASH) [53] and Survodutide [54] phase 2 randomized trials

Drug	% MASH resolution without worsening of fibrosis	EDP % [95% CI]	% with ≥ 1 grade improvement in fibrosis without worsened steatohepatitis	EDP % [95% CI]
Tirzepatide (SYNERGY-NASH) (52 weeks) (*p* < 0.001 for all comparisons)				
5 mg	44	34 [17;50]	55	25 [5;46]
10 mg	56	46 [29, 62]	51	22 [1;42]
15 mg	62	53 [36;69]	51	21 [1;42]
Placebo	10	-	30	-
Survodutide (48 weeks) (*p* < 0.001 for all comparisons)				
2.4 mg	47	33	34	Not available
4.8 mg	62	48	36	Not available
6 mg	43	29	34	Not available
Placebo	14	-	22	-

*MASH* Metabolic dysfunction associated steatohepatitis, *EDP* Estimated difference in responder populations, *95% CI* 95% confidence interval

## Adverse effects of the incretin class of drugs

Gastrointestinal intolerances (i.e., nausea, vomiting and changes in bowel habits) are the most common side effects reported and are related to delayed gastric emptying and stimulation of neural emetogenic centers. While typically mild to moderate in severity, they represent a significant driver for premature drug discontinuation and will have implications for therapeutic longevity. Emphasis on slow dose titration, small frequent meals, adequate hydration, moderation of dietary fat intake and adjunctive use of anti-emetics can help with therapy adherence.

The use of incretin drugs may precipitate gallstone disease. Cholelithiasis is also common after bariatric surgery, suggesting a relationship with rapid weight loss and alterations in eating habits which may promote cholestasis [55]. Heightened awareness of the potential for clinically significant cholelithiasis events is warranted when patients are on these agents.

Incretin therapies are also associated with elevations in serum amylase and lipase levels. However, the pathologic significance of this phenomenon is unclear. There have been infrequent reports of fatal acute pancreatitis and these agents should be used with caution (or avoided) in people with a history of clinically significant pancreatitis. Similarly, long acting GLP1 agonists carry FDA black box warnings for medullary thyroid C-cell cancer based on pre-clinical models but these are ultimately, rare clinical events. A large retrospective cohort study suggested GLP1 receptor agonist use was not associated with a substantially increased risk (1.33 events per 10,000 person years) of thyroid cancer over a mean follow-up of 3.9 years [56]. Conversely, Bezin et al. found use of GLP-1 receptor agonists was associated with an increased risk of all thyroid cancer (adjusted hazard ratio [HR] 1.58, 95% CI 1.27–1.95) and for medullary thyroid cancer (adjusted HR 1.78, 95% CI 1.04–3.05) [57]. These drugs are currently contraindicated if there is a history of thyroid cancer or multiple endocrine neoplasia (MEN) syndromes.

## Incretin drugs in MAFLD/MASH: ready for prime time?

While GLP1 receptor agonists have demonstrated utility in MASH, whether this translates into discernible improvements in major liver-related clinical outcomes (i.e., cirrhosis and clinical decompensation, transplant-free survival, HCC etc.) remains to be elucidated. The emphasis on hard clinical endpoints is pertinent given the high cost of these drugs and lifelong adherence to therapy demanded of patients. In this regard, results from phase 2 of the ESSENCE study which examines the impact of Semaglutide in MASH on clinical liver outcomes (Due 2027) is highly anticipated.

The MASH treatment pipeline is becoming increasingly competitive with multiple agents entering the trial space with positive data (Table 3). What differentiates these therapies from one another and which drug will prove the most useful remains to be elucidated. Clearly, there are mechanistic and pharmacokinetic considerations that will likely become apparent as additional phase 3 and head-to-head comparison studies are conceptualized. At current, it is difficult to make direct comparisons between agents due to the variable placebo response rates seen in trials. This is likely due to participants being prescribed health interventions by their treating physicians when diagnosed with a “lifestyle” disease. Standardizing trial design with a wash in period of behavioral change before randomization may help to clarify true treatment effects. This would also be consistent with real world practice where treatments are not siloed and often integrated with lifestyle changes. Similarly, expanding trials to include patients with co-factors for liver disease (alcohol and viral hepatitis) which are commonly encountered in clinical practice would emphasize the external validity of therapeutic efficacy. Recent retrospective data suggest that the use of GLP1 receptor agonists may reduce rates of hepatic decompensation in patients with MAFLD and alcohol-related liver disease [58].

**Table 3:** Key biopsy-based clinical trials of incretin analogs in metabolic dysfunction associated steatohepatitis (MASH)

GLP 1 receptor agonists				MASH resolution without worsening of fibrosis?	Fibrosis regression (≥ 1 grade) without worsening of steatohepatitis?
NCT01237119	Liraglutide (1.8 mg once daily subcut) vs placebo (2016) (Phase 2) [51]	MASH with F0-4 fibrosis	n = 52	Yes (p = 0·019)	-
NCT02970942	Semaglutide (0.1, 0.2 or 0.4 mg subcut) vs. placebo (2021) (Phase 2) [52]	MASH with F1-3 fibrosis	n = 320	Yes (p < 0.001)	No (p = 0.48)
NCT03987451	Semaglutide (2.4 mg once weekly subcut) vs. placebo (2023) (Phase 2) [59]	MASH cirrhosis	n = 71	No (p = 0.29)	No (p = 0.087)
NCT04822181	Semaglutide (2.4 mg once weekly subcut) vs placebo (2024) (ESSENCE) (Phase 3) [48]	MASH with F2-3 fibrosis	n = 800	Yes (p < 0.001)	Yes (p < 0.001)
NCT05016882	Semaglutide with FGF-21 or Amylin analog combination therapy vs placebo (Phase 2)	MASH	Recruiting	Pending	Pending
NCT04639414	Semaglutide with empagliflozin vs empagliflozin vs placebo (COMBATT2NASH) (Phase 4)	MASH	Recruiting	Pending	Pending
NCT03648554	Dulaglutide vs placebo (REALIST) (Phase 4)	MASH	Recruiting	Pending	Pending
GLP1/GIP receptor dual agonists					
NCT04166773	Tirzepatide (5, 10 or 15 mg once weekly subcut) vs placebo (SYNERGY-NASH) (Phase 2) [53]	MASH with F2-3 fibrosis	n = 190	Yes (p < 0.001)	Yes (p < 0.001)
GLP1/Glucagon receptor dual agonists					
NCT04771273	Survodutide (2.4, 4.8, or 6.0 mg once weekly subcut) vs placebo (Phase 2) [54]	MASH with F1-3 fibrosis	n = 293	Yes (p < 0.001)	Yes (p < 0.001)
NCT05877547	Efinopegdutide vs Semaglutide vs placebo (Phase 2)	MASH with F2-3 fibrosis	Recruiting	Pending	Pending
NCT05989711	Pemvidutide vs placebo (IMPACT) (phase 2)	MASH with F2-3 fibrosis	Recruiting	Pending	Pending
GLP1/GIP/Glucagon triple agonists					
NCT04505436	Efocipegtrutide vs placebo (Phase 2)	MASH with F1-3 fibrosis	Recruiting	Pending	Pending

It is estimated that up to a third of patients prescribed GLP1 receptor agonists stopped treatment after 1 year in some jurisdictions [60]. Cost is certainly a barrier (and may be less so in countries with universal health coverage), but more data on adherence patterns is required to formulate mitigation strategies. Non-persistence with incretin drugs might become a public health concern if formulations and indications for use continue to be expanded on. Premature cessation is associated with significant weight re-gain, presumably in the same composition to which it was lost in clinical trials. The impact of this rebound physiology upon end organ function is unclear at this stage but will have important ramifications for drug coverage and patient selection.

Few patients with cirrhosis were included in previous studies of the incretin-mimetics and this represents an unmet need in the field. Loomba et al., demonstrated that once weekly subcutaneous Semaglutide was not superior to placebo in remitting inflammation and fibrosis in a small (*n* = 71) cohort of patients with MASH and compensated cirrhosis [59]. However, the drug was well tolerated and associated with improvements in the cardiometabolic risk profile and fibrogenesis markers. Despite the disappointing primary outcome, the initial safety signals in chronic liver disease are reassuring.

Given the discordant outcomes between cirrhosis and earlier fibrosis stages of the MAFLD/MASH spectrum, there is a need to develop tools that more precisely phenotype patients and identify those most likely to experience benefits with incretin drugs. Clarity around dosing principles particularly in those with significant fibrosis but who are of healthy weight, whether complete remission of liver fibrosis (i.e., a F0 state) is possible with monotherapy, and the duration of treatment required also needs to be clarified.

MASH and fibrosis staging has historically required liver biopsy, but this is invasive, costly, and not feasible at a population level. Non-invasive tests are a potential solution but for acceptance as a decision-making tool will need to fulfill the dual prerequisites of being able to both approximate the histologic grading of steatohepatitis and staging of fibrosis (per clinical trial entry criteria) and ultimately, also predict liver related clinical outcomes. To this end, there are several permutations combinations of imaging and/or blood biomarker-based tools (acFibroMASH Index, FibroScan-aspartate aminotransferase [FAST]), MAST (MRI-AST), MEFIB (magnetic resonance elastography [MRE] plus FIB-4) and FNI (fibrotic NASH index) and others in the developmental pipeline (e.g., N3-MASH panel) that have entered the trial arena with promising data [61–64]. Active incorporation of these tests into trial designs (and their validation in real world practice) are required to promote clinical applicability of results. Machine learning algorithms are tantalizing prospects to further improve the performative aspects of these modalities but remain speculative at the current time [65]. Non-invasive tools to monitor therapy response and define futility endpoints is another unmet need. This is difficult in the case of GLP1-based therapies, which have proven efficacy as systemic metabolic regulators with pleiotropic effects on end-organ health. Focusing on single organ endpoints will run anathema to the concept of holistic patient care.

As the mechanisms underpinning MAFLD disease progression become more clearly defined, clinically significant molecular signatures that may modulate treatment responses should also be adapted into therapeutic paradigms.

Indeed, Ding et al. have leveraged multiomic technologies to define three distinct subtypes of MAFLD in a Chinese cohort that may predict inflammatory burden and the development of clinically significant endpoints such as HCC and cirrhosis [66]. Ethnically clustered single nucleotide polymorphisms (SNPs) in the patatin-like phospholipase domain-containing protein 3 (PNPLA3) gene are also associated with more aggressive disease, even in the absence of classic metabolic risk factors (i.e., the “lean MAFLD” phenotype) [67]. Variants in the PNPLA3 genotype attenuate the anti-inflammatory effects of Tirzepatide on MASH resolution [68]. Importantly, whether lean MAFLD patients experience similar degrees of weight loss to their obese/overweight counterparts and the safety signals related to possible iatrogenic sarcopenia are unclear. Such patients with isolated fatty liver disease (a minority) may be better suited to treatment with liver-directed pharmacotherapies. Only Resmetirom (a thyroid hormone receptor beta agonist) has FDA approval for this indication although there are several other drugs in the pipeline. Finessing patient selection and effectively integrating pharmacotherapies into clinical workflows will be a priority in the coming years. Acknowledging the complex multi-hit pathogenesis of MASH, it is reasonable to speculate that permutations of incretin therapy with other agents targeting diverse mechanisms of action may prove to be the best strategic approach to tailor therapy to individual patient phenotypes in future. Given the nascent nature of clinical trial data in the MASH arena, additional studies looking at subtypes of MASH participant phenotypes, drug combinations, dosing regimens etc. will need to be conceptualized to provide firm guidance in this area.

Sarcopenia and frailty are major prognostic determinants in cirrhotic, multimorbid and elderly patients. Weight loss inevitably leads to a reduction in lean mass and is an important consideration when using any form of bariatric therapy, including the incretin analogs. However, the absolute quantum of lean mass loss is quite variable, reflecting trial design, drug dosing, and pharmacokinetics. Moreover, whether this observed phenomenon of lean mass loss translates into an exacerbation of the frailty phenotype is unclear. Lean body mass is a composite measure that encapsulates the sum of muscle, organs, and fluid content. Hence decrements in lean mass observed with incretin therapies may not reflect clinically significant declines in muscle bulk [69]. Moreover, it is postulated that the improvements in insulin signaling may promote adaptive responses that reduce muscle steatosis and produce more functionally robust myocytes. Recent trials with Liraglutide [70] and Tirzapetide [71] support the notion that despite the reductions in lean muscle mass observed, this is not pathologically disproportionate to the degree of weight loss achieved. Moreover, magnetic resonance imaging assessments suggest significant reductions in muscle fat infiltration that produce higher quality muscle composition overall. Longer term frailty outcomes data is required to lend credence to these hypotheses. Nevertheless, strategies to mitigate muscle loss during bariatric interventions are important. In this regard, the incorporation of regular resistance exercise is a simple strategy to improve both muscle quality and reduce frailty [72]. Optimizing dietary protein intake and micronutrient supplementation is also pertinent given that patients experience significant appetite suppression and weight loss on incretin drugs. Hypercatabolic disease states like cirrhosis add additional layers of complexity for managing nutrition. Defining optimal nutritional targets and assessing the role of nutraceuticals like branched chain amino acids (BCAAs) in promoting anabolism is thus important. Adjunctive approaches focusing on the pharmacologic augmentation of muscle mass represent another tantalizing prospect for positive body habitus recomposition.

## Conclusion

The fatty liver diseases are a major public health concern and reflects the end organ manifestation of a much more sinister problem, that of underlying systemic metabolic dysfunction. Incretin drugs demonstrate multiple benefits on organ physiology and hold promise to transform the treatment landscape of the CKLM disease axis as the backbone medical therapy. Gastrointestinal side effects are common but can be managed with the appropriate support. Further work on defining the impact on clinical outcomes, risk mitigation, combination therapeutics and treatment-response biomarkers are required to finesse use of these agents in MAFLD/MASH, especially in MAFLD-related cirrhotic patients.

## Data Availability

Not applicable.
